# Ingestion of *Bt* corn pollen containing Cry1Ab/2Aj or Cry1Ac does not harm *Propylea japonica* larvae

**DOI:** 10.1038/srep23507

**Published:** 2016-03-23

**Authors:** Yanmin Liu, Qingsong Liu, Yanan Wang, Xiuping Chen, Xinyuan Song, Jörg Romeis, Yunhe Li, Yufa Peng

**Affiliations:** 1 State Key Laboratory for Biology of Plant Diseases and Insect Pests, Institute of Plant Protection, Chinese Academy of Agricultural Sciences, Beijing, 100193, China; 2 Jilin Academy of Agricultural Sciences, Changchun, Jilin, 130124, China; 3 Agroscope, Institute for Sustainability Sciences ISS, 8046 Zurich, Switzerland

## Abstract

*Propylea japonica* (Thunberg) (Coleoptera: Coccinellidae) is a prevalent pollen consumer in corn fields and is therefore exposed to insecticidal proteins contained in the pollen of insect-resistant transgenic corn cultivars expressing Cry proteins derived from *Bacillus thuringiensis* (*Bt*). In the present study, the potential effect of Cry1Ab/2Aj- or Cry1Ac-containing transgenic *Bt* corn pollen on the fitness of *P. japonica* larvae was evaluated. The results show that the larval developmental time was significantly shorter when *P. japonica* larvae were fed pollen from *Bt* corn cultivars rather than control pollen but that pupation rate, eclosion rate, and adult fresh weight were not significantly affected. In the feeding experiments, the stability of the Cry proteins in the food sources was confirmed. When *Bt* corn pollen passed through the gut of *P. japonica*, 23% of Cry1Ab/2Aj was digested. The results demonstrate that consumption of *Bt* corn pollen containing Cry1Ab/2Aj or Cry1Ac has no detrimental effect on *P. japonica* larvae; the shortened developmental time of larvae that consumed these proteins was likely attributable to unknown differences in the nutritional composition between the *Bt*-transgenic and control corn pollen.

Corn (*Zea mays* L.) is the most important grain crop in the world, and in 2014, 21.5 million tons of corn were produced in China^1^. Corn yields are significantly reduced by insect pests and especially by *Ostrinia furnacalis* (Guenée) (Lepidoptera: Crambidae), which is commonly known as the Asia corn borer. The species is estimated to cause about 10% yield loss of corn grain each year, but losses can be as high as 30% with heavy infestations^2^. In addition, injury caused by *O. furnacalis* on corn can increase the severity of diseases, such as stalk rot and ear rot, and lower the quality of corn grain^3^.

In 1993, Koziel *et al.* reported that genetically engineered (GE) corn plants expressing an insecticidal protein derived form *Bacillus thuringinesis* (*Bt*) experience reduced damage from *Ostrinia nubilalis* (Hübner) (Lepidoptera: Crambidae)^4^. Since then, various *Bt* corn cultivars expressing *Bt* insecticidal genes such as *cry1Ab, cry1Ac, cry1F* and *cry3Bb1* have been developed and are commercially grown in many countries^5^. China has also devoted great effort to develop *Bt* corn plants that are resistant to *O. furnacalis*; to date, many *Bt* corn cultivars expressing *cry1Ab, cry1Ac, cry1Ah* , *cry 1Ab/2Aj* or *cry1Ah/cry1Ie* have been obtained^6^,^7^,^8^,^9^,^10^,^11^,^12^,^13^, and laboratory and field tests have shown that some of these *Bt* corn cultivars exhibited high resistance to the target pest. This includes the cultivars SK12-5 (Shuangkang12-5) (expressing a *cry 1Ab/2Aj fusion gene)* and BT-799 (expressing a modified *mcry1Ac* gene) that have entered the preproduction testing stage, and thus may be commercially grown in China in the near future.

Although insect-resistant GE crops represent an efficient form of crop protection, they may pose potential risks to the environment, and therefore any new GE plant must be subjected to a rigorous risk assessment before it is commercially planted. An important component of the risk assessment concerns potential effects on non-target beneficial arthropods^14^,^15^,^16^,^17^,^18^.

The ladybird beetle, *Propylea japonica* (Thunberg) (Coleoptera: Coccinellidae), is a common and abundant natural predator in many crop and non-crop systems throughout East Asia^19^,^20^,^21^. Both larvae and adults of *P. japonica* are predaceous, feeding predominantly on aphids but, also on planthoppers, whiteflies, and eggs and young larvae of lepidopterans^22^,^23^. During plant anthesis, and especially when insect prey is scarce, *P. japonica* adults and larvae also feed on plant pollen, including corn pollen^24^,^25^. Therefore, *P. japonica* has the potential to be exposed to plant-produced insecticidal proteins by feeding on herbivores in *Bt* crop fields but also by feeding on *Bt* corn pollen.

In the present study, we investigated the potential effects of consumption of *Bt* corn pollen containing Cry1Ab/2Aj or Cry1Ac protein on *P. japonica* larvae in the laboratory.

## Results

### Pollen consumption by *P. japonica* larvae

**Figure 1:**
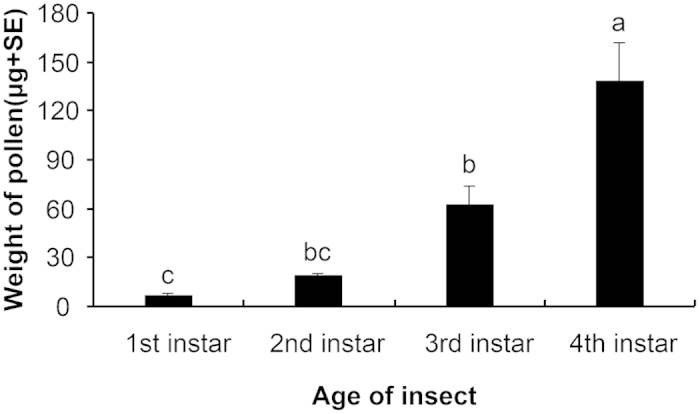
Consumption of corn pollen by *Propylea japonica* larvae (μg per individual per day). The larvae (7 to 10 per instar) were fed exclusively with Z58 corn pollen for 1 day. Values are means + SE. Means with different letters are significantly different at P < 0.05 according to a one-way ANOVA followed by a Tukey HSD test.

Larvae of *P. japonica* readily consumed maize (Z58) pollen. Pollen consumption increased significantly with the growth and development of *P. japonica* larvae from the first to fourth instar (one-way ANOVA: *F* = 22.8, *P* < 0.001) (Fig. 1). The dry weight (DW) (mean ± SE) of a single Z58 pollen grain was estimated to be 274.2 ± 8.1 ng.

### Effects of *Bt* corn pollen on life table parameters of *P. japonica*

**Figure 2:**
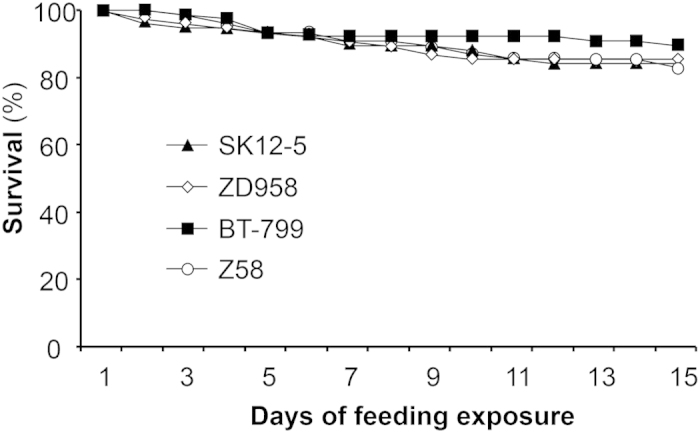
Survival of *Propylea japonica* larvae that were fed pollen from one of two *Bt* corn cultivars or from the corresponding non-transformed cultivars. Larvae were fed a combination of soybean aphids and pollen from: SK12-5 expressing Cry1Ab/2Aj; the control for SK12-5 (ZD958); BT-799 expressing Cry1Ac; and the control for BT-799 (Z58) (n = 76).

Over 70% of the *P. japonica* larvae that exclusively consumed corn pollen developed to adults, and the percentage did not differ for larvae that fed on *Bt* corn pollen vs. those that fed on the corresponding control pollen (χ^2^-test; both *P* > 0.05) (Table 1). Similarly, larval survival did not significantly differ for larvae that fed on *Bt* corn pollen vs. those that fed on the the corresponding control pollen (Cry1Ab/2Aj: χ^2^ = 0.07, *P* = 0.79; Cry1Ac: χ^2^ = 0.09, *P* = 0.77) (Fig. 2). However, larval developmental time was significantly shorter for larvae that fed on *Bt* corn pollen than for larvae that fed on the corresponding control pollen (Mann-Whitney *U*-test; Cry1Ab/2Aj: *U* = 1602.5, *P* = 0.034; Cry1Ac: *U* = 1568.0, *P* = 0.010). Adult weight did not significantly differ for larvae that fed on *Bt* corn pollen vs. those that fed on the the corresponding control pollen (Student’s *t*-test; Cry1Ab/2Aj: *t* = 0.07, *df* = 64, *P* = 0.95 for females and *t* = −0.40, *df* = 45, *P* = 0.69 for males; Cry1Ac: *t* = 0.64, *df* = 67, *P* = 0.52 for females and *t* = −0.33, *df* = 48, *P* = 0.74 for males).

### Stability of Cry proteins in *Bt* corn pollen

According to our double–antibody sandwich enzyme-linked immunosorbent assays (DAS-ELISA), the original concentrations (mean ± SE) of Cry1Ab/2Aj in SK12-5 pollen and of Cry1Ac in BT-799 pollen were 22.8 ± 1.8 μg/g and 0.059 ± 0.0033 μg/g DW, respectively. After a 2-day feeding exposure in the environmental chamber, the Cry1Ab/2Aj content decreased (without statistical significance) to 18.7 ± 1.5 μg/g DW (Student’s *t*-test; *t* = 1.74, *df* = 8, *P* = 0.12), while the Cry1Ac content significantly decreased to 0.036 ± 0.004 μg/g DW (Student’s *t*-test; *t* = 4.97, *df* = 8, *P* = 0.001). No *Bt* protein was detected in pollen from the control corn cultivars (ZD958 and Z58).

### Fate of Cry1Ab/2Aj in *Bt* corn pollen after larval gut passage

After passage through the gut of *P. japonica* larvae, the pollen grains of SK12-5 contained 23.21% less Cry1Ab/2Aj protein than the fresh pollen. However, this difference was not statistically significant (Student’s *t*-test; *t* = 0.73, *df* = 4, *P* = 0.50) (Table 2). A comparable analysis with pollen from BT-799 was not conducted because the Cry1Ac concentration in the pollen was very low, probably because of the low level activity of the constitutive CaMV35s promoter in pollen.

## Discussion

To evaluate the potential effect of *Bt* corn pollen on a non-target organism using a dietary exposure assay, it first needs to be established that the test species readily accepts the corn pollen provided^26^. Results from the pollen consumption assay in the current study indicated that the *P. japonica* larvae readily accepted and utilized corn pollen. As the larvae developed and grew, the amount of corn pollen that they consumed steadily increased, and at the fourth instar a single larva contained an average of 500 pollen grains in its gut (equivalent to about 138 μg DW). In a previous study in which *P. japonica* larvae were fed rape seed pollen, Zhang *et al.*^27^ reported that a single fourth instar of *P. japonica* larva contained about 15000 pollen grains in its gut which was equivalent to about 150 μg DW pollen. This difference can be explained by the difference in pollen size, i.e., corn pollen grains are nearly 30-times larger and heavier than rape seed pollen grains^27^,^28^. Fourth-instar larvae of *P. japonica* collected in corn fields were found to contain fewer than 200 pollen grains in their body (unpublished data), indicating that the *P. japonica* larvae have been exposed to more *Bt* corn pollen under laboratory conditions in the current study than under the field conditions.

The results of our feeding assays indicated that survival and development of *P. japonica* larvae were not detrimentally affected when they fed on transgenic corn pollen containing Cry1Ab/2Aj or Cry1Ac. The larval developmental time, however, was slightly (less than 4%) but significantly reduced when larvae were fed corn pollen containing either Cry1Ab/2Aj or Cry1Ac rather than control corn pollen. In our previous study, in contrast, the developmental time was significantly prolonged when *P. japonica* larvae were fed *Bt* rice pollen containing Cry1C or Cry2A protein, even though extensive experimentation demonstrated that *P. japonica* was not affected when fed a rapeseed pollen-based diet containing purified Cry1C or Cry2A at concentrations that were >10-times higher than in *Bt* rice pollen^28^. Although the reasons for the changes in developmental time are unclear, we suspect that the changes are due to unknown differences in the nutritional composition of the *Bt* pollen^28^,^29^. The active part of Cry1Ab/2Aj contained in *Bt* corn pollen is similar to Cry1Ab (Z. C. Shen, personal communication), and a previous study confirmed that *P. japonica* is not sensitive to either Cry1Ab or Cry1Ac^27^. Therefore, the “positive” effect of *Bt* corn pollen containing Cry1Ab/2Aj or Cry1Ac on *P. japonica* developmental time in the current study is unlikely to be due to the action of Cry proteins contained in the *Bt* corn pollen. Furthermore, this minor effect on developmental time may not affect *P. japonica* population dynamics in the field, because *P. japonica* will consume much less *Bt* corn pollen in the field than in our laboratory assays. The pollen shedding period of corn usually lasts only 5–8 days with a maximum of 14 days^30^, which is shorter than the duration of pollen feeding in our laboratory experiments. In addition, *P. japonica* in the field will consume other foods in addition to *Bt* corn pollen.

To clarify the exposure level of *P. japonica* larvae to Cry1Ab/2Aj and Cry1Ac our laboratory feeding experiments, we determined the stability of both Cry proteins in the food source (corn pollen). ELISA results indicated that the Cry1Ab/2Aj and Cry1Ac concentrations in the *Bt* corn pollen decreased by 20–40% during the 2-day feeding exposure. In a previous field study, the content of Cry1Ab protein in *Bt* corn pollen rapidly declined to 38.6% of the initial concentration after the pollen was shed and accumulated in the leaf axil^31^. Moreover, the activity of the Cry proteins in corn pollen may be reduced by exposure to rainfall and sunlight before the pollen is consumed by *P. japonica* larvae in the field. Therefore, although the Cry protein contents in *Bt* corn pollen decreased by up to 40% during the feeding experiments, the larvae in our bioassays were likely exposed to much higher Cry protein concentrations than larvae in the field.

Because corn pollen grains are not completely digested by *P. japonica*, we considered the possibility that most of the Cry protein in the pollen may be excreted with feces without contacting the larva’s gut tissue and thus without the opportunity to bind to the gut membrane, which is a prerequisite for Cry protein toxicity^32^. Based on our quantification of Cry protein concentration in *Bt* corn pollen before and after gut passage, over 76% of the Cry1Ab/2Aj in *Bt* corn pollen was excreted with feces; this further suggests that the absolute exposure of *P. japonica* larvae to Cry proteins in *Bt* corn pollen is likely to be extremely low. In contrast, over 60% of Cry proteins were digested when *Bt* corn pollen passed though the digestive system of adults of *Chrysoperla carnea* (Stephens) (Neuroptera: Chrysopidae)^32^.

Because biological control is important for insect pest management, a number of studies have assessed the potential non-target effects of *Bt* crop pollen on *P. japonica*. Although a previous study reported that feeding on Cry1Ah-containing corn pollen affected the activity of some gut enzymes of *P. japonica*^33^, a follow-up study confirmed that consumption of Cry1Ah-containing corn pollen did not significantly affect the growth or development of *P. japonica*^34^. Bai *et al.*^19^ reported that consumption of *Bt* rice pollen containing Cry1Ab did not reduce the fitness of *P. japonica*. Using a dietary exposure assay, another study confirmed that *P. japonica* is not sensitive to Cry1Ab, Cry1Ac, Cry1F, Cry1C, or Cry2A, which are expressed by a number of different crop plants^27^,^28^. In addition, Bai *et al.* reported that the development of *P. japonica* fed with *Nilaparvata lugens* (Stål) (Hemiptera: Delphacidae) that had been reared on Cry1Ab-contained *Bt* rice plants was not affected^35^.

Overall, the present study shows that the consumption of *Bt* corn pollen expressing Cry1Ab/2Aj or Cry1Ac does not reduce the fitness of *P. japonica* larvae. The decreased developmental time for *P. japonica* larvae when fed *Bt* corn pollen may be attributed to the altered nutritional composition of the *Bt* corn pollen caused by gene transformation or other unknown factors. Moreover, this effect seems to be positive rather than negative in that it benefitted the growth of the larvae. Finally, we suspect that this minor effect is unlikely to be detectable in the field, because *P. japonica* larvae use pollen only as a supplementary food and thus consume much less corn pollen in the field than in the current laboratory experiment. Therefore, we conclude that the growing of Cry1Ab/2Aj- and Cry1Ac-transgenic corn should pose a negligible risk to *P. japonica.*

## Materials and Methods

### Insects

Specimens of *P. japonica* were collected in 2014 at the experimental field station of the Institute of Plant Protection, Chinese Academy of Agricultural Sciences (CAAS), near Langfang City, Hebei Province, China (39.5°N, 116.7°E). A colony was subsequently maintained in the laboratory without introduction of field-collected insects for over two generations. Both larvae and adults of *P. japonica* were reared on soybean seedlings infested with *Aphis glycines* Matsumura (Hemiptera: Aphididae). The aphids were replaced daily, ensuring *ad libitum* food for the developing larvae. Newly hatched *P. japonica* larvae (<12 h after emergence) were used for the experiments. The insects were kept in a climatic chamber at 26 ± 1 °C, 75 ± 5% RH and a 16:8 h light: dark photoperiod.

### Corn plants and pollen collection

The transgenic *Bt* corn cultivars SK12-5 (Shuangkang12-5) and BT-799 and their corresponding non-transformed near isolines, ZD958 (Zhengdan 958) and Z58 (Zheng 58), respectively, were used in the experiments. SK12-5 plants express a *cry1Ab/2Aj* fusion gene, and BT-799 plants express a modified *mCry1Ac* gene; these genes encode for Cry proteins that target lepidopteran corn pests including *O. furnacalis*. Expression of the *cry1Ab/2Aj* gene is driven by the constitutive pZmUbi-1 promoter, and expression of the *mCry1Ac* gene is driven by the constitutive CaMV35s promoter. The seeds of SK12-5 and ZD958 were provided by Professor Zhicheng Shen (Zhejiang University, Hangzhou, China), and the seeds of BT-799 and Z58 were provided by Professor Jinsheng Lai (China Agricultural University, Beijing, China).

The corn cultivars were simultaneously cultivated at the experimental field station of Jilin Academy of Agriculture Sciences in Gongzhuling City, Jilin Province, China (43°19′N, 124°29′E) in 2014. The plants were grown in 12 adjacent plots (plot size: 400 m^2^, 20 m × 20 m) in a completely randomized design (3 plots per cultivar). The corn seeds were sown on 25 May 2014. The plants were cultivated according to the common local agricultural practices without pesticide sprays.

During corn anthesis in late July 2014, corn pollen was collected by shaking the corn tassels in a plastic bag. The collected pollen was air dried at room temperature for 48h and subsequently passed through a screen (0.2-mm openings) to remove anthers and contaminants. Pollen collected from each corn cultivar was pooled and stored at −60 °C until further use. The concentrations of the fusion protein Cry1Ab/2Aj and the Cry1Ac proteins in pollen were determined using ELISA as described below.

### Feeding system for *P. japonica*

The pollen-based diet used in the current study was developed in our previous study^27^ and has been successfully used to assess the potential effect of *Bt* rice pollen on *P.japonica.*^27^,^28^ The *P. japonica* larvae were individually confined in Petri dishes (6.0 cm diameter, 1.5 cm height) and fed with corn pollen on the first day of each instar and were then provided with a mixture of corn pollen and soybean aphids until they developed into the next instar. The pollen was directly sprinkled on the bottom of the dish, while the aphids were provided on 2-cm segments of heavily infested soybean seedlings. Pollen was replaced every 2 days, and soybean aphids were replaced daily. In addition, an open 1.5-ml centrifuge tube containing solidified 1% agar solution was added to each feeding container as a water source. All of the food elements were provided *ad libitum*.

### Pollen consumption by *P. japonica* larvae

To determine whether *P. japonica* larvae readily accept corn pollen and to determine the number of pollen grains consumed during the feeding experiment, pollen from the non-*Bt* corn cultivar Z58 was fed to *P. japonica* using the feeding system described in the previous section. For each larval stage, 7–10 larvae were collected after feeding exclusively on pollen for 1 day. All larvae were then frozen at −20 °C. To count the pollen grains in the larval gut, the larvae were thawed and dissected, and the entire alimentary canal (gut) was excised from each specimen. Subsequently, the gut was transferred to a 1.5-ml centrifuge tube containing 100 μl of carbolic acid dye solution. After the gut was ruptured with a thin needle, a vortex mixer was used to mix the pollen suspension. An aliquot (5 μl) of the suspension was transferred to a glass slide, and pollen grains (including full, partially digested, and empty grains) were counted with a microscope at 50× magnification. Three 5-μl aliquots were taken from each insect gut preparation and counted. The mean number of pollen grains in the aliquots was multiplied by 20 to obtain the number in a single larval gut^32^.

To calculate the weight of pollen in the gut of *P. japonica*, the mean weight of a single pollen grain was estimated. Lyophilized fresh corn pollen (1.0 mg) was mixed with 300 μl of carbolic acid dye solution. The pollen grains were counted in each of three 5-μl aliquots of the suspension with a microscope at 50× magnification. The mean number of pollen grains in the aliquots was multiplied by 60 to obtain the number in the whole sample. Finally, the weight of each sample (1.0 mg) was divided by the number of grains to obtain the mean DW of an individual pollen grain. In this process, three samples were measured. Based on the mean individual DW of pollen grains and the number of pollen grains found in the larval guts, the mean weight of pollen in the gut of *P. japonica* was calculated^32^.

### Effect of *Bt* corn pollen on *P. japonica*

An experiment was conducted in which *P. japonica* was fed *Bt* or non-*Bt* (control) corn pollen using the feeding system described above. There were four pollen treatments with 76 neonates per treatment: (i) SK12-5 corn pollen containing Cry1Ab/2Aj; (ii) ZD958 corn pollen (the non-*Bt* control pollen for SK12-5); (iii) BT-799 corn pollen containing Cry1Ac; and (iv) Z58 corn pollen (the non-*Bt* control pollen for BT-799). The experiment was conducted in an environmental chamber at 26 ± 1 °C and with 75 ± 5% RH and a 16:8 h light: dark photoperiod. Larval survival and developmental time were recorded twice per day (9:00 am, 9:00 pm). When adults emerged, they were individually weighed with an electronic balance (CPA224S; Sartorius AG; readability = 0.1 mg, repeatability <± 0.1 mg).

To determine the temporal stability of the *Bt* proteins in the corn pollen during the experiment, five pollen subsamples were taken from both *Bt* corn cultivars before and after being exposed to *P. japonica* for 2 days. The Cry protein concentrations in pollen samples were determined by ELISA as described later in the Methods.

### Fate of Cry1Ab/2Aj in *Bt* corn pollen after ladybird gut passage

To estimate the degree to which *P. japonica* larvae were exposed to Cry protein when *Bt* corn pollen grains passed through their gut, the mean Cry1Ab/2Aj contents in pollen grains before ingestion and after ingestion (in the larval feces) were compared.

Neonates of *P. japonica* were fed SK12-5 corn pollen until the third instar using the feeding system described above and then starved for 24 h to empty their gut. The starved larvae were then placed in Petri dishes (6.0 cm diameter, 1.5 cm height) (three dishes with three larvae per dish) and exclusively provided with SK12-5 corn pollen. Their fresh fecal pellets were collected at 3-h intervals three times during the first 12 h after the larvae had been placed in the dishes. All feces collected from each Petri dish were pooled as one sample, resulting in a total of three samples. The samples were stored at −80 °C. After the samples were lyophilized, the concentrations of Cry1Ab/2Aj were measured using ELISA as described below.

The total digestion rate of Cry1Ab/2Aj in pollen cannot be determined based on *Bt* protein concentrations (Cry protein per dry weight of pollen) because the digestion process reduces both the amount of Cry protein and the weight of the pollen grains. Therefore, the mean Cry protein content of a single pollen grain before ingestion or present in the feces was evaluated. With the method described in the section “Pollen consumption by *P. japonica*”, the mean dry weight of an individual pollen grain from fresh pollen or feces was calculated. Based on the individual dry weight of pollen grains and the *Bt* protein concentrations in fresh corn pollen and feces, the Cry1Ab/2Aj protein content in single pollen grains was estimated^32^.

### ELISA measurements

The concentrations of the fusion protein Cry1Ab/2Aj and the protein mCry1Ac in corn pollen or *P. japonica* feces were determined using ELISA kits from EnviroLogix (Portland, ME; catalog number AP 003). The kits were originally designed for quantitative detection of Cry1Ab and Cry1Ac. Based on the recommendation from the laboratory where the fusion gene was produced (Z. C. Shen, personal communication), the fusion protein Cry1Ab/2Aj can be quantified using this ELISA kit. The same Cry1Ab calibrators could be used for developing the standard curve, but the concentrations were adjusted to the equivalent Cry1Ab/2Aj concentrations of 0, 1.0, 5.0, and 10.0 ppb because the molecular weight of Cry1Ab/2Aj is two-times that of Cry1Ab. The ELISA kit could be directly used for detection of mCry1Ac with the same Cry1Ab calibrators, but the concentrations were adjusted to the equivalent Cry1Ac concentrations of 0, 1.5, 10.0, and 25.0 ppm. Five samples (2 to 5 mg each) of *Bt* or control corn pollen were lyophilized and mixed with 1 ml of PBST buffer (provided in the kit) with a micro-mortar and pestle on ice. After centrifugation and appropriate dilution of the supernatants, ELISA was performed according to the manufacturer’s instructions. The optical density (OD) values were read with a microplate spectrophotometer (PowerWave XS2, BioTek, USA). The concentrations of Cry1Ab/2Aj and Cry1Ac were calculated by comparing the OD values to the standard curves.

### Statistical analyses

Pollen consumption was compared among *P. japonica* instars using a one-way ANOVA followed by a Tukey HSD test. In the experiment in which *P. japonica* larvae were fed *Bt* corn pollens or the corresponding non-*Bt* corn pollens, pair-wise statistical comparisons were made between each *Bt* corn pollen and the corresponding non-*Bt* corn pollen. Chi-square tests were used to compare pupation rates and eclosion rates. Mann-Whitney *U*-tests were used to compare larval developmental times because the data did not satisfy the assumptions for parametric analyses (normal distribution of residuals and homogeneity of error variances). The effects of dietary treatments on *P. japonica* survival were analyzed with the Kaplan-Meier procedure and Logrank test.

Student’s *t*-tests were used to compare Cry protein concentrations in fresh *Bt* corn pollen and in pollen that had been exposed to larvae for 2 days. Student’s *t*-tests were also used to compare adult weights and the mean Cry1Ab/2Aj content in corn pollen grains before and after gut passage.

All statistical analyses were conducted using the software package SPSS (version 13; SPSS, Inc., Chicago, IL, USA).

## Additional Information

**How to cite this article**: Liu, Y. *et al.* Ingestion of *Bt* corn pollen containing Cry1Ab/2Aj or Cry1Ac does not harm *Propylea japonica* larvae. *Sci. Rep.* **6**, 23507; doi: 10.1038/srep23507 (2016).

## Figures and Tables

**Table 1:** Effect of consumption of pollen from *Bt* corn (SK12-5 containing Cry1Ab/2Aj and BT-799 containing Cry1Ac) or from the corresponding non-transformed cultivars (ZD958 and Z58) on life table parameters of *Propylea japonica*.

Parameter	Corn cultivar			
	SK12-5 (Cry1Ab/2Aj)	ZD958	BT-799 (Cry1Ac)	Z58
Pupation rate (%)^a^	82.89 (76)	84.21 (76)	89.47 (76)	81.58 (76)
Eclosion rate (%)^b^	75.00 (76)	73.68 (76)	78.95 (76)	77.63 (76)
Days to pupa ( ± SE)	9.87 ± 0.19 (63)*	10.11 ± 0.16 (64)	9.76 ± 0.10 (68)*	10.14 ± 0.12 (62)
Female fresh weight (mg ± SE)	6.57 ± 0.16 (31)	6.55 ± 0.18 (35)	6.31 ± 0.09 (37)	6.19 ± 0.18 (32)
Male fresh weight (mg ± SE)	5.38 ± 0.12 (26)	5.45 ± 0.14 (21)	5.16 ± 0.16 (23)	5.24 ± 0.19 (27)

Number of replicates is indicated in parentheses.

Each *Bt* corn cultivar was compared to its corresponding control. An asterisk indicates a significant difference between a *Bt* corn cultivar and its control (P < 0.05) according to χ^2^ test for pupation and eclosion rates, Mann-Whitney U–test for days to pupation, ans Student’s *t*-test for fresh weights.

^a^The percentage of larvae that advanced to the pupa stage.

^b^The percentage of larvae that advanced to the adult stage.

**Table 2:** Cry1Ab/2Aj protein content of corn pollen grains before and after passage through the digestive system of *Propylea japonica* larvae.

Sample	Cry1Ab/2Aj concentration (μg/g dry weight)	No. of pollen grains per mg	Cry1Ab/2Aj content per grain (pg)^a^	Rate of Cry1Ab/2Aj protein loss (%)^b^
Fresh pollen	22.09 ± 2.74 (a_1_)	4300.84 ± 28.28 (b_1_)	5.14 ± 0.67 (c_1_)	23.21
Feces	8.49 ± 3.12 (a_2_)	2157.35 ± 14.65 (b_2_)	3.95 ± 1.48 (c_2_)	

Feces were collected from larvae that were fed SK12-5 corn pollen; n = 3.

Values in columns 2 to 4 are means ± SE.

^a^Calculated as follows: c_x_ = a_x_/b_x_ × 10^3^.

^b^Calculated as follows: R_Cry_ = (c_1_-c_2_)/c_1_ × 100.
